# De novo transcriptome sequencing and analysis of salt-, alkali-, and drought-responsive genes in *Sophora alopecuroides*

**DOI:** 10.1186/s12864-020-06823-4

**Published:** 2020-06-23

**Authors:** Fan Yan, Youcheng Zhu, Yanan Zhao, Ying Wang, Jingwen Li, Qingyu Wang, Yajing Liu

**Affiliations:** College of Plant Science, Jilin University, 5333 Xi’an Road, Changchun City, Jilin Province China

**Keywords:** *Sophora alopecuroides*, Salt, Alkali, Drought, Transcriptome, Differentially expressed genes, Illumina sequencing

## Abstract

**Background:**

Salinity, alkalinity, and drought stress are the main abiotic stress factors affecting plant growth and development. *Sophora alopecuroides* L., a perennial leguminous herb in the genus *Sophora*, is a highly salt-tolerant sand-fixing pioneer species distributed mostly in Western Asia and northwestern China. Few studies have assessed responses to abiotic stress in *S. alopecuroides*. The transcriptome of the genes that confer stress-tolerance in this species has not previously been sequenced. Our objective was to sequence and analyze this transcriptome.

**Results:**

Twelve cDNA libraries were constructed in triplicate from mRNA obtained from *Sophora alopecuroides* for the control and salt, alkali, and drought treatments. Using de novo assembly, 902,812 assembled unigenes were generated, with an average length of 294 bp. Based on similarity searches, 545,615 (60.43%) had at least one significant match in the Nr, Nt, Pfam, KOG/COG, Swiss-Prot, and GO databases. In addition, 1673 differentially expressed genes (DEGs) were obtained from the salt treatment, 8142 from the alkali treatment, and 17,479 from the drought treatment. A total of 11,936 transcription factor genes from 82 transcription factor families were functionally annotated under salt, alkali, and drought stress, these include *MYB*, *bZIP*, *NAC* and *WRKY* family members. DEGs were involved in the hormone signal transduction pathway, biosynthesis of secondary metabolites and antioxidant enzymes; this suggests that these pathways or processes may be involved in tolerance towards salt, alkali, and drought stress in *S. alopecuroides*.

**Conclusion:**

Our study first reported transcriptome reference sequence data in *Sophora alopecuroides,* a non-model plant without a reference genome. We determined digital expression profile and discovered a broad survey of unigenes associated with salt, alkali, and drought stress which provide genomic resources available for *Sophora alopecuroides*.

## Background

At present, it is an indisputable fact that global climate has changed, posing a potential threat to the sustainable development of agriculture and food security [1]. Increasing global temperatures cause sea level to rise, which in turn increases the salinity of groundwater in coastal and arid areas [1]. Salt-alkali land is widely distributed around the world, covering about 100 million hectares [2]. According to the Food and Agriculture Organization of the United Nations, more than 400 million hectares of land on the major continents are affected by salt [3]. Rising salinization may reduce agricultural acreage by up to 20% per year by 2050 [4]. The amount of land in India that has been degraded by excess sodicity and salinity is estimated to be about 6.75 million hectares [5]. In China, there are about 100 million hectares of salinized land [6]. Drought, which can cause salinity to increase, has a great impact on crop yield [7, 8]. With the changes in global climate, the frequency and duration of drought events is increasing, with serious impacts on crop yields [9, 10]. To solve the growing global food shortage, it is essential to use saline-alkali land for agriculture. Using effective gene resources to cultivate salt-, alkali-, and drought-resistant crops is the most economical and productive measure to solve this problem.

Plant breeding and gene transformation are important ways to improve crop tolerance to abiotic stress. Stress-regulatory mechanisms in higher plants have been analyzed by researching many of the genes related to abiotic-stress tolerance at the transcriptional level [11, 12]. Various signal transduction pathways are involved in plant responses to abiotic stress; these include the phospholipid signaling pathway [13], calcium-dependent protein kinase pathway, mitogen-activated protein kinase cascade pathway [14], and abscisic acid (ABA) pathway [15]. These pathways form a signal transduction network by which plants respond to abiotic stress [16]. In addition, stress tolerance mechanisms include a series of gene expression and gene product interactions, which enhance plant adaptations to abiotic stress at cellular and molecular levels [17]. Many differentially expressed genes (DEGs), which encode reactive oxygen species scavenging proteins, aquaporins, heat shock proteins, and ion transporters have been identified in stress resistance [18].

Technological developments have provided great convenience in biological science research. Next-generation sequencing of RNA, which can directly determine cDNA sequences, has been widely used to identify plant genes [19, 20]. RNA-seq and DEG-analysis have revealed mechanisms of responses to complex biotic and abiotic stressors in many plant species [21], including *Arabidopsis thaliana* [22], *Vitis vinifera* [23], *Ammopiptanthus mongolicus* [24], *Cucumis sativus* [25], and *Gossypium hirsutum* [26]. For instance, in *A. thaliana*, about 30% of the transcriptome is considered to be involved in abiotic-stress regulation, and 2409 genes have been identified as being of great importance in drought resistance, salt tolerance, and resistance to cold [27, 28].

*Sophora alopecuroides L.* (Fabaceae) is a highly stress-tolerant leguminous perennial herb in the genus *Sophora*, distributed mainly in western Asia and northwestern China [29, 30]. *Sophora alopecuroides* is an important potential resource for stress resistance genes. However, few studies have focused on finding stress resistance genes in *S. alopecuroides* using the transcriptome sequencing method. In this study, we perform transcriptome sequencing of *S. alopecuroides* plants subjected to three stress treatments (salt, alkali, and drought). We use de novo sequence assembly and differential gene expression analysis, and screen many genes related to abiotic resistance. Our study provides new genetic resources for research of abiotic resistance in crop plants, thereby increasing the options for genetic crop improvements.

## Results

*Sophora alopecuroides* is a type of perennial herb and drought tolerant plant, with its drought tolerance closely related to its root system. Using the method of saline, alkaline and drought treatments used on *Arabidopsis* and soybean, it was found that when concentrations of NaCl, NaHCO_3_ and PEG were more than 1.2, 1.2 and 8% respectively, the growth of *Sophora alopecuroides* was inhibited or the plant wilted (Fig. S1). After 72 h, plant growth and physiological indexes were significantly different and relatively stable. Therefore, we used 1.2% NaCl, 1.2% NaHCO_3_ and 8% PEG-treated *Sophora alopecuroides* roots as tissues for constructing a cDNA library for transcriptome sequencing, from which differences in gene expression under saltine, alkaline and drought conditions could be explored.

### Transcriptome sequencing and assembly

**Fig. 1:**
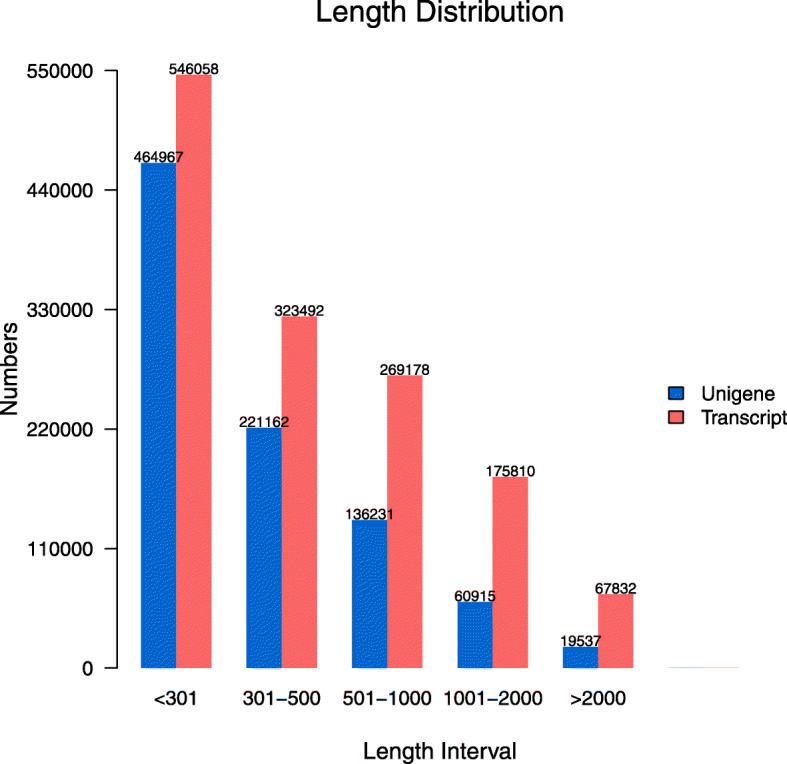
De novo assembly length distribution of sequences for *Sophora alopecuroides*. Transcripts: red; Unigenes: blue

In total, 605,800,814 raw sequencing reads were obtained from the control and treated samples. And 586,189,628 clean reads were used to gather the data (Table 1). 1,382,370 transcripts and 902,812 unigenes were obtained, with an average length of 366 bp and 294 bp, respectively. Six hundred eighty-six thousand one hundred twenty-nine unigenes were 200 to 500 bp long, and 80,452 were > 1000 bp long (Fig. 1).

**Table 1:** Summary of *Sophora alopecuroides* sequences analyzed

Sample	Raw Reads	Clean reads	Clean bases	Error (%)	Q20 (%)	Q30 (%)	GC (%)
CK_1	47,691,636	46,202,320	6.93G	0.01	97.79	94.15	44.01
CK_2	41,826,612	40,474,310	6.07G	0.01	97.90	94.42	43.73
CK_3	50,190,638	49,125,200	7.37G	0.01	97.61	93.93	43.91
ST_1	46,150,030	44,279,092	6.64G	0.01	97.32	93.51	43.22
ST_2	50,369,242	48,508,750	7.28G	0.01	97.82	94.37	43.51
ST_3	54,517,670	52,656,970	7.90G	0.01	97.85	94.40	43.48
A_ST_1	48,586,426	47,023,258	7.05G	0.01	97.67	93.94	46.67
A_ST_2	58,315,134	56,447,032	8.47G	0.01	97.63	93.90	46.23
A_ST_3	53,214,668	51,438,942	7.72G	0.01	97.54	93.68	44.09
DT_1	57,566,808	55,738,546	8.36G	0.01	97.76	94.20	43.46
DT_2	49,791,136	48,134,912	7.22G	0.01	97.91	94.42	43.59
DT_3	47,580,814	46,160,236	6.92G	0.01	97.92	94.42	43.62
Summary	605,800,814	586,189,568	87.93G				

The numbers 1–3 after CK, ST and A_ST, and DT identify the three independent biological replicates for the control and salt, alkali, and drought treatments, respectively

Q20: The percentage of bases with a Phred value > 20

Q30: The percentage of bases with a Phred value > 30

### Functional annotation of all non-redundant unigenes

The annotated number unigenes for each database is shown in Table 2. Among the unigenes, 357,522 (39.6%) had significant matches in the Nr database (NCBI redundant protein Sequences), 214,419 (23.75%) in the Nt database (NCBI nucleotide Sequences), and 293,553 (32.51%) in the Swiss-Prot database. Among the 902,812 unigenes, 545,615 (60.43%) had at least one highly match with an identified gene in BLAST searches (Table 2).

**Table 2:** BLAST analysis of non-redundant unigenes sequenced for *Sophora alopecuroides*, against public databases

	Number of Unigenes	Percentage (%)
Annotated in Nr	357,522	39.6
Annotated in Nt	214,419	23.75
Annotated in KO	157,394	17.43
Annotated in Swiss-Prot	293,553	32.51
Annotated in PFAM	356,271	39.46
Annotated in GO	366,814	40.63
Annotated in KOG	178,137	19.73
Annotated in all Databases	47,161	5.22
Annotated in at least one Database	545,615	60.43
Total Unigenes	902,812	100

*Nr* NCBI non-redundant protein Sequences, *Nt* NCBI nucleotide Sequences, *Pfam* Protein family, *KOG/COG* KOG: euKaryotic Ortholog Groups; COG: Clusters of Orthologous Groups of proteins, *Swiss-Prot* A manually annotated and reviewed protein sequence database, *KEGG* Kyoto Encyclopedia of Genes and Genomes, and *GO* Gene Ontology

### Functional classification by GO and KOG

**Fig. 2:**
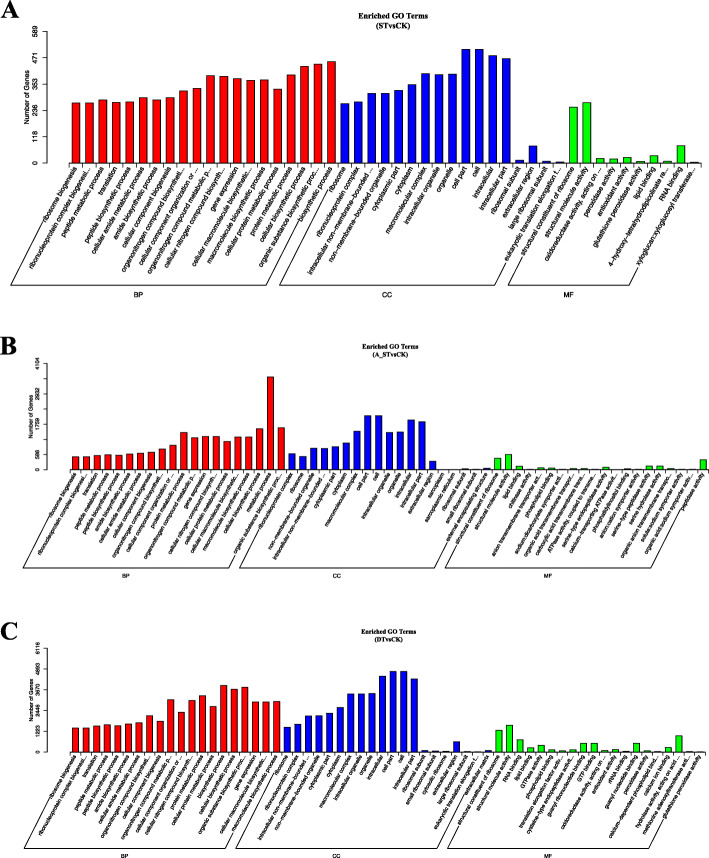
Histogram of GO classification for *Sophora alopecuroides*. The results are summarized in three main categories: Biological Process, Cellular Component, and Molecular Function. The x-axis indicates the subcategories, and the y-axis shows the number of genes associated with the GO terms. The subset “ST&CK” (panel **a**) indicates the number of DEGs between the salt treatment and control, “A_ST&CK” (panel **b**) the number of DEGs between the alkali treatment and control, and “DT&CK” (panel **c**) the number of DEGs between the drought treatment and control

The GO (Gene Ontology) classification that we used includes three main classes of ontology. The salt-, alkali-, and drought-treatment samples were examined by GO functional significant enrichment analysis. For the salt-treatment samples, 1178 DEGs were annotated into 47 categories; 5863 DEGs were annotated into 59 categories for the alkali-treatment samples; and 2232 DEGs annotated into 60 categories for the drought-treatment samples. In the biological process, the most enriched categories in salt- and drought-treatment were the biosynthetic, organic substance biosynthetic, and cellular biosynthetic processes. In contrast, in the alkali-treatment samples, the most enrichment occurred in the metabolic process category. In the cellular component, the most enrichment occurred in relation to cellular morphology, cell, and intracellular. In the molecular function, the most enrichment occurred in relation to structural constituents of ribosome, structural molecule activity, and molecular function (Fig. 2).

**Fig. 3:**
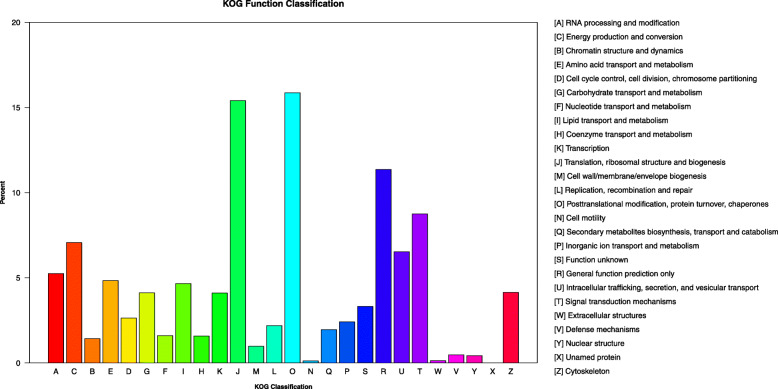
Functional classification of the assembled unigenes for *Sophora alopecuroides*. The y-axis indicates the percentage of genes annotated relative to all the annotated genes

One hundred seventy-eight thousand one hundred thirty-seven unigenes were annotated to 26 groups in KOG database. Among these groups, the largest were those involved in protein turnover, post-translational modification, and chaperones (28271), followed by translation, ribosomal structure, and biogenesis (27448), general function prediction (20248), signal transduction mechanisms (15600). Few unigenes relate to groups involved in cell motility (223) and extracellular structures (245) (Fig. 3).

### Functional classifications using KEGG pathways under salt stress

All unigenes were annotated and mapped to KEGG database (http://www.genome.jp/kegg/). Of the 1673 DEGs sequenced from the salt-treatment samples, 616 were annotated to 61 metabolic pathways (Table S1). Of these, 64 up-regulated DEGs were annotated in 25 of the metabolic pathways, and 552 down-regulated genes were annotated in 55 of the metabolic pathways. In these 61 metabolic pathways, only 6 were annotated to the up-regulation of DEGs, and 19 metabolic pathways annotated both up-regulated and down-regulated genes. Only 36 metabolic pathways annotated down-regulated genes.

In the process of plant secondary metabolite synthesis, the coenzyme A gene, *SaCoA,* involved in the phenylpropanoid biosynthesis pathway (ko00940) and phenylalanine metabolism (ko00360), corresponds to the upregulation of differential genes under salt stress. Coenzyme A is an important cofactor in many biosynthesis, degradation, and energy generation pathways [31]. Previous studies have shown that the coenzyme A biosynthesis enzyme phosphoryltransferase participate in plant growth, salt resistance, and osmotic stress [32]. The coenzyme A biosynthetic pathway corresponds to regulating plant salt tolerance [33]. In the study of *Zygophyllum* spp., it was found that under salt stress conditions, the CoA contents of the salt-tolerant varieties in the control group and the salt-treated group did not differ significantly, whereas the CoA contents of the salt-sensitive varieties decreased significantly [33]. In the ABA signaling pathway, *SaPYL4–1*, *SaPYL4–2*, *SaPYL4–3*, *SaPYL4–4,* and *SaPYL5–1* were found to be related to ABA receptors (Table S2). These five genes were down-regulated both under salt and alkali treatments. Four upregulated DEGs, *SaPP2C8*, *SaPP2C16*, *SaPP2C37,* and *SaPP2C53*, were related to protein phosphatase 2C (Table S2). Moreover, *SaPP2C8* and *SaPP2C53* also showed up-regulation under alkali stress. This result is consistent with the confirmed relationship between *PYL* and *PP2C* in the ABA signaling pathway [34]. In the signaling pathway of the plant hormone brassinolide, a gene *SaTCH4* related to xyloglucosyl transferase TCH4 was identified under salt stress. This gene is involved in the regulation of cell elongation, which may be related to the suppression of plant growth under salt stress conditions [35].

### Functional classifications using KEGG pathways under alkali stress

Of the 8142 DEGs sequenced from the alkali-treatment samples, 2644 DEGs were annotated to 118 metabolic pathways in the KEGG database. Of these, 2056 up-regulated DEGs were annotated in 117 metabolic pathways, and 588 down-regulated DEGs were annotated in 64 metabolic pathways. Among these 118 metabolic pathways, there were only 54 metabolic pathways that contained up-regulated DEGs. There were 63 metabolic pathways that annotated both up-regulated and down-regulated DEGs, whereas only one metabolic pathway annotated down-regulated genes.

Under alkali stress, the positive regulatory gene *SaNPR1* was obtained in the signal transduction pathway. The *SaNPR1* gene was annotated as a regulatory protein NPR1-like, which is a positive regulatory gene in the salicylic acid signal pathway. Studies have shown that *NRP1* participates in abiotic stresses including low temperature and salt stress through the salicylic acid signaling pathway [36–40], and that salicylic acid, as a plant stress signal, plays an important role under high pH stress conditions [41]. In the ethylene signaling pathway, 17 genes related to serine/threonine-protein kinase CTR1 were upregulated. These included *SashkB, SashkC1*, *SashkC2*, *SashkC3*, *SashkC4, SakinX*, *SadrkD*, *SagefX*, *SaDDB1*, *SaDDB2*, *SaDDB3*, *SaDDB4*, *SaDDB5, SaMIMI1*, *SaMIMI2, Sapats1–1,* and *Sapats1–2* (Table S2). Studies have confirmed that CTR1 was a positive factor regulating abiotic stress [42]. Six negative regulatory genes were obtained in the auxin signaling pathway, including the auxin influx carrier (AUX1 LAX family) gene *SaAUX1*, and auxin-responsive protein IAA genes *SaIAA8–1*, *SaIAA8–2*, *SaIAA14*, *SaIAA26,* and *SaIAA27* (Table S2). These genes are mainly involved in cell enlargement and plant growth. The histidine kinase 4 (cytokinin receptor) gene *SaAHK4*, which is involved in cytokine signaling pathway, also exhibits negative regulation under drought stress. In the gibberellin signaling pathway, the negatively regulated DELLA protein GAI-like gene *SaGAI* was obtained, which was mainly involved in plant stem growth and induction of germination. In the brassinolide signaling pathway, the negatively regulated D3-type cyclin isoform 1 gene, *SaCYCD3,* was obtained, which was mainly involved in the cell division process. The negative regulatory genes we obtained are mainly involved in the growth and development of plants [43–52]. Previous studies in *Arabidopsis* have found that plants can further improve their resistance to stress by slowing down growth and promoting leaf senescence [34]. Furthermore, we obtained two up-regulated genes in the plant secondary metabolite synthesis pathway, namely *SaGCDH* and *SaOMT6*. The caffeoyl-CoA O-methyltransferase gene is related to the *Citrus reticulata* flavonoid biosynthesis process, and flavonoids, as the primary secondary metabolites, play a crucial part in plant stress resistance [42]. The up-regulated differential genes related to antioxidant enzymes mainly include *SaPXR1*, superoxide dismutase genes (*SaSOD1, SaSOD2–1, and SaSOD2–2*), and the putative peroxisomal-coenzyme A synthetase gene *SaHACL1*. These genes are mainly involved in the removal of ROS responding to stress, and thus reduce the damage of plant cells and tissues by reactive oxygen species [53, 54].

### Functional classifications using KEGG pathways under drought stress

Of the 17,479 DEGs sequenced from the drought-treatment samples, 6546 DEGs were assigned to 121 metabolic pathways; of these, 576 up-regulated DEG annotations were in 101 metabolic pathways, and 5970 down-regulated DEG annotations were in 120 metabolic pathways. Of the 121 metabolic pathways, one was annotated to up-regulated DEGs, and 100 contained both up-regulated and down-regulated genes; the other 20 annotated only down-regulated genes.

Under drought stress, multiple positively regulated expression genes were obtained in the phytohormone signal transduction pathway and found to participate in the ABA signaling pathway. Four genes, namely *SaPYL4–1*, *SaPYL4–2*, *SaPYL5,* and *SaPYL9*, were found to be related to the abscisic acid receptor PYR/PYL family (Table S2). In a study on Arabidopsis abiotic stress, it was found that ABA receptor-related genes are accompanied by up-regulation of ABA to activate the Arabidopsis stress resistance system, which is a positive regulator of Arabidopsis adaptation to abiotic stress [34]. In this process, PYL-related genes promote the expression of serine/threonine-protein kinase gene by inhibiting the expression of PP2C-related genes [34]. Further analysis revealed four serine/threonine-protein kinase genes (*SaSRK2e-1*, *SaSRK2e-2*, *SaSRK2e-3,* and *SaSAPK1*) and one ABA response element-binding protein 1 gene (*SaABF1*). These genes are consistent with the expression pattern of Arabidopsis ABA in response to stress [42, 55]. Therefore, we presumed that the up-regulated expression of these genes may responsible for the drought tolerance of *Sophora alopecuroides*. In addition, we obtained four negative regulatory genes involved in plant hormone signal transduction pathways, including SAUR-like auxin-responsive family protein (*SaSAUR32*), sensory histidine protein kinase (*SaAHK2* and *SaAHK4*), and histidine-containing phosphotransfer protein 1 (*SaAHP1*), which are mainly involved in cell growth, cell division, bud germination, and plant growth. Studies have confirmed that under drought stress, Arabidopsis can respond to adversity stress by weakening its growth [42, 55, 56]. We speculate that there is a similar mechanism in *Sophora alopecuroides*, and the downregulated expression of genes *SaSAUR32*, *SaAHK2*, *SaAHK4,* and *SaAHP1* is a stress response. In secondary metabolite synthesis-related pathways, we identified upregulated genes, including shikimate O-hydroxycinnamoyl transferase (*SaHST*), catalase isozyme 1 (*SaCAT1–1*, *SaCAT1–2*, *SaCAT1–3*, *SaCAT1–4,* and *SaCAT1–5*), and peroxisome biogenesis protein 5 (*SaPEX5*) (Table S2). These genes are mainly involved in the active oxygen scavenging mechanism. After the plant is subjected to adversity stress, it will be accompanied by secondary stress damage, such as that by reactive oxygen species. In response to oxidative stress, plants form peroxidase, superoxide dismutase, and catalase, which are used to remove active oxygen species and reduce the damage caused by them to plant cells [53, 54].

### Analysis of differential gene expression

**Fig. 4:**
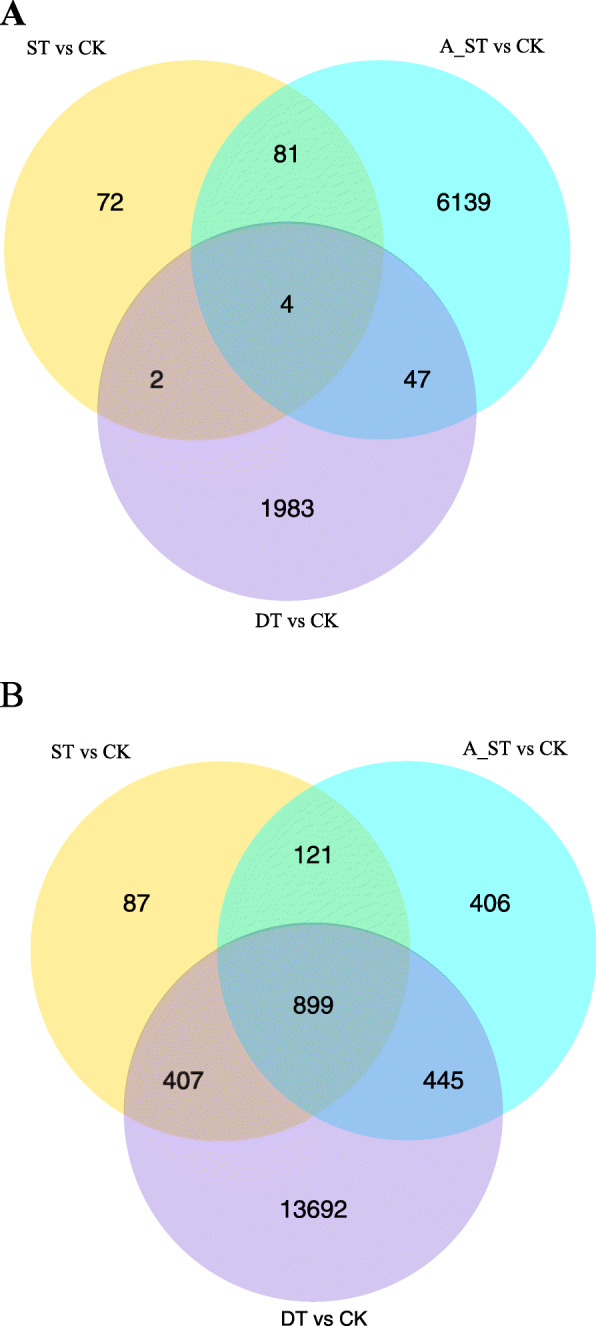
Venn diagram of DEGs sequenced for *Sophora alopecuroides*. The sum of the numbers in each large circle represents the total number of DEGs between combinations. The overlapping part of the circles represents DEGs for the treatment combinations. “ST&CK”: number of DEGs between salt treatment and control; “A_ST&CK”: number of DEGs between alkali treatment and control; “DT&CK”: number of DEG between drought treatment and control. **a**: up-regulation of DEGs; **b**: down-regulation of DEGs

The number of DEGs under saline, alkaline and drought conditions was quite different (Fig. 4). The number of DEGs in the drought treatment was 17,479, of which 2036 were upregulated and 15,443 were downregulated. In the alkaline treatment 8142 DEGs were obtained, of which 6271 were upregulated and 1871 were downregulated. There were only 1673 DEGs under saline stress conditions, with 159 upregulated and 1514 downregulated. Of all the DEGs, four were upregulated in all 3 treatments and 899 were downregulated.

Furthermore, 11,936 DEGs were annotated into 82 transcription factor families, including 814 *MYB* transcription factors, 472 *bZIP* transcription factors, 166 *WRKY* transcription factors, and 123 *NAC* transcription factors (Table S3). There were 42 *MYB* transcription factors corresponding to 6 KEGG metabolic pathways, and 25 *bZIP* transcription factors corresponding to 7 KEGG metabolic pathways. However, one *NAC* transcription factor corresponded to only one KEGG metabolic pathway, and 25 *WRKY* transcription factors corresponded to 6 KEGG metabolic pathways.

Among the differential genes, three MYB transcription factors, *SaMYB1*, *SaMYB5,* and *SaMYB14,* were identified up-regulating under salt and alkali stress (Table S2). *SaMYB1* belongs to LHY (K12133) of the circadian rhythm – plant pathway (Ko04712) in the KEGG pathway analysis. *SaMYB5* corresponds to CDC5 (K12850) of the spliceosome pathway (Ko03040). In addition, we screened the *SaMYBG* gene, which showed negative regulation under drought and alkali conditions. Previous results suggested that overexpression of soybean MYB transcription factors *GmMYBJ1*, *GmMYBJ2*, *GmMYB12B2*, *GmMYB3a,* and *GmMYB68* increased the tolerance of Arabidopsis to salt, alkali, and drought [57–61]. *AtMYB11*, *AtMYB12,* and *AtMYB111* of *Arabidopsis thaliana* have different regulatory effects on flavonoid biosynthesis [62–64]. We speculate that *SaMYB14*, *SaMYB1*, *SaMYB5,* and *SaMYBG* might also have similar regulatory functions under salt, alkali, and drought stress.

Many bZIP transcription factor genes have been identified in different plant species, such as cucumbers, corn, and legumes [65–67]. In plants, bZIP transcription factors are essential for various biological processes, such as seed maturation, stress signal transduction, and flower development [68]. Arabidopsis *AtbZIP17* and *AtbZIP28* are involved in regulating root elongation during stress response [69]. In this study, we screened 3 bZIP transcription factor genes: *SabZIP1,* upregulated under alkali stress; *SabZIP3,* upregulated under salt stress; and *SabZIP5,* downregulated under both salt and drought stress (Table S2). Furthermore, *SabZIP1* was involved in ko00280, ko00270, ko00260 and ko00250 pathway.

WRKY transcription factors response to stress by regulating plant secondary metabolites, such as alkaloids, terpenes, and other subclasses [70, 71]. In this study, three WRKY transcription factor genes were obtained: *SaWRKY27*, *SaWRKY33,* and *SaWRKY38* (Table S2). Both *SaWRKY27* and *SaWRKY33* were upregulated under alkali stress and participated in the plant-pathogen interaction (Ko04626) pathway. *SaWRKY38* was downregulated under both alkali and drought stress conditions. In common bean, 8 up-regulated WRKY transcription factors were confirmed under drought stress [71]. Most WRKY transcription factors obtained in *Dunaliella bardawil* can bind to the W-box and response to n abiotic stress [72]. In peanut, 73 differentially expressed *AhWRKY* genes affected by drought stress were obtained through expression pattern analysis of the WRKY gene family [73]. *SbWRKY30* enhanced the drought tolerance of sorghum by regulating the drought stress response gene *SbRD19* [74]. The overexpression of the wheat WRKY transcription factor gene *TaWRKY13* can significantly improve salt tolerance in rice, indicating that the WRKY transcription factor was a positive factor responding to salt stress [75].

We analyzed NAC transcription factors and found that *SaNAC2*, *SaNAC5,* and *SaNAC25* were upregulated under alkali stress and drought stress (Table S2). Studies in celery (*Apium graveolens* L.) have shown that *AgNAC63* (a homologous gene of ANAC072/RD26) is highly induced under the conditions of heat, cold, and salt [76]. Through transcriptome analysis of 4 cotton varieties, it was determined that 120 NAC transcription factor genes may be involved in response to salt, alkali and drought stress [77]. *MfNACs* in *Medicago falcate* maintain glutathione levels by regulating the gene expression of *glyoxalase* 1, which is a defense response against drought stress [78].

### Idenfication of the differentially expressed genes (DEGs)

**Fig. 5:**
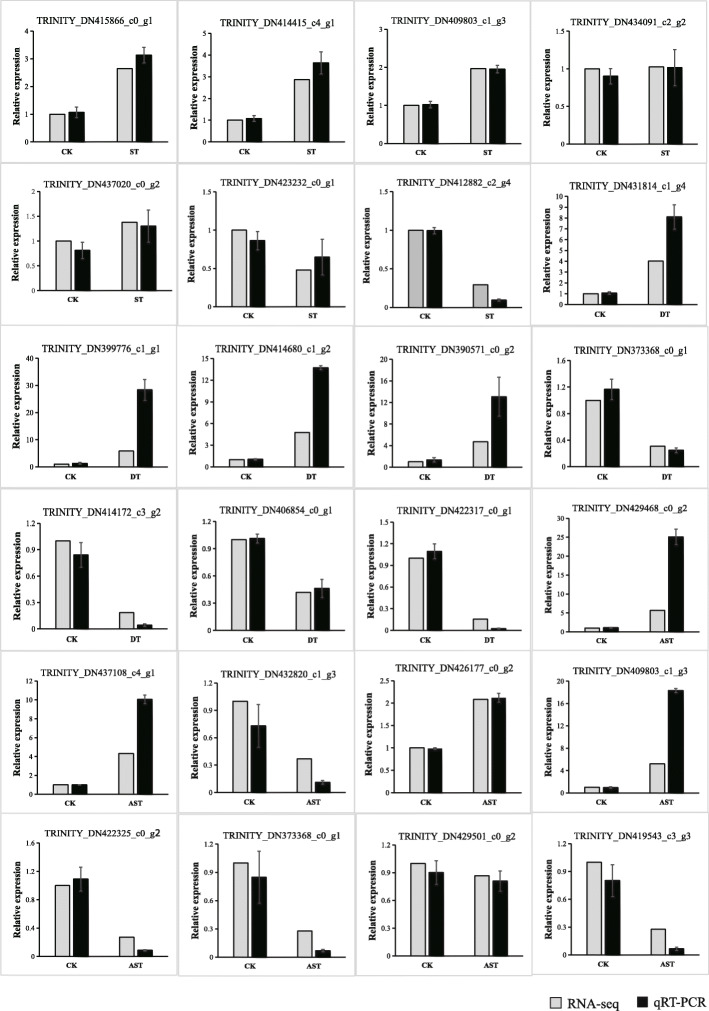
The relative expression levels of representative DEGs between control and stress-treatment samples, sequenced for *Sophora alopecuroides*. Actin was used as internal reference. Relative transcription levels were calculated using the 2^−ΔΔCt^ method. Data represent means ± standard deviation (SD) from three biological replicates and three technical replicates. RNA-seq value is base on fold chang of upregulation or downregulation DEGs. The relative expression level indicates the fold change obtained by quantitative RT-PCR. Values are the means ±SD. Means were generated from three independent replicates. Statistical comparisons (one-way ANOVA) are presented for each variable (** *P* < 0.01; * *P* < 0.05)

To confirm that the DEGs obtained by Illumina Hiseq 2000 and 2500 platform sequencing were credible, 24 DEGs (13 up-regulated and 11 down-regulated) were randomly chosen for qRT-PCR analysis (Fig. 5 and Table S4). The results showed that expressions of these DEGs were similar to those obtained by RNA-seq. The results indicate that the method used to confirm DEG in this experiment is feasible.

## Discussion

*Sophora alopecuroides* (known as ku dou zi in Chinese medicine) is famous for its tolerance to high salinity, alkalinity and drought. The genome of *S. alopecuroides* has not been sequenced previously. We therefore analyzed the transcriptome sequence of *S. alopecuroides* under different stress treatments, explored the changes in DEGs and metabolic pathways under salt, alkali, and drought stress, and searched for genes related to abiotic-stress resistance. This study focuses on the expression of genes in the roots of Sophora alopecuroides, which has some limitations. Many genes (DEGs) were expressed differentially between the treatments and control. We found many transcription factor genes, including *MYB, bZIP, NAC, ERF* and *WRKY* family members, which were functionally annotated in all three treatments. Transcription factor families (MYB, WRKY, NAM, NAC, ATAF, AP2-EREBP, bHLH, C2H2, HSF, ERF, and bZIP) have been found responsible for abiotic stress in rice [79–84], maize [82], *A. thaliana* [83], soybean [84] and tobacco; overexpression of these TFs enhanced stress tolerance in transgenic plants [79, 83]. In this study, 4 MYB TFs (*SaMYB14*, *SaMYB1*, *SaMYB5*, and *SaMYBG*), 3 bZIP TFs (*SabZIP1*, *SabZIP3*, and *SabZIP5*), 3 WRKY TFs (*SaWRKY27*, *SaWRKY33*, and *SaWRKY38*), and 3 NAC TFs (*SaNAC2*, *SaNAC5*, and *SaNAC25*) were screened out as potential candidate genes that respond to salt, alkali, and drought stress (Table S2).

Significant enrichment analysis showed that the DEGs, mainly those related to plant membrane binding and signal transduction, were basically consistent with those of cotton [26, 85, 86], *Caragana korshinskii* [87], and banana [88, 89]. In the salt and drought treatments, the most abundant DEG types were those involved in biosynthesis. In the alkali treatment, the most abundant DEGs were those involved in metabolic processes. The most abundant DEGs involved in molecular functions and producing cellular components were the same under all three treatments.

By cross-analysis of DEGs obtained under the three treatments, we found that only four of the up-regulated DEGs were shared among the three treatments. Among the down-regulated DEGs, those that were common to the three treatments were mainly related to plant signal transduction and metabolism, including the ABA signaling pathway, peroxisome and flavonoid biosynthesis. ABA, as an important plant hormone, plays a key role in plant growth and development, including seed germination, reproduction, and seed dormancy. ABA also plays an important role in plant responses to biological and abiotic stress, particularly salt, drought, and cold stress [26, 85–91]. Transcription factor *ATHB6* is involved in down-regulating ABA responses, for instance during seed germination and stomatal closure, when plants are sensitive to ABA [92]. However, the up-regulated DEG *SaATHB* annotation obtained in this study is related to the homeobox-leucine zipper protein, reflecting differences in DEG responses to stress under different treatments. The results need to be validated prospectively.

The results of GO enrichment analysis showed that there were differences in DEGs and metabolic pathways between the salt-, alkali-, and drought-stress samples, indicating that the response mechanisms of plants to salt, alkali, and drought stress were not identical under different treatments. Hormone signal transduction pathway is a KEGG pathway directly related to plant metabolism. This pathway involves zeatin biosynthesis, tryptophan metabolism, diterpenoid biosynthesis, carotenoid biosynthesis. Nine DEGs were involved in carotenoid biosynthesis under salt treatment. Among these, five downregulated DEGs (*SaPYL4–1*, *SaPYL4–2*, *SaPYL4–3*, *SaPYL4–4,* and *SaPYL5–1*) were related to the pyrabactin resistance 1-like (PYR/PYL) ABA receptor, and four upregulated DEGs (*SaPP2C8*, *SaPP2C16*, *SaPP2C37,* and *SaPP2C53*) were related to protein phosphatase 2C (PP2C). Furthermore, five *SaPYLs* and two *SaPP2Cs (SaPP2C8* and *SaPP2C53)* showed the same expression pattern between salt and alkali stress. This result indicates that there is a negative regulation between PYR/PYL and PP2C, which is same as previous studies that showed that PYR/PYL inhibits PP2C [34, 93]. Under drought treatment the expression of PYL-related genes in the ABA signaling pathway was different from that under saline-alkali stress. Four PYL-related genes, *SaPYL4–1*, *SaPYL4–2*, *SaPYL5*, and *SaPYL9* were up-regulated under drought stress, but were down-regulated under salt and alkali. The finding indicated that the mechanisms of drought stress and saline-alkali stress responses is different in *Sophora alopecuroides*.

In addition, under salt treatment conditions, a down-regulated gene *SaTCH4* was identified in the brassinosteroid biosynthesis pathway, specifically in xyloglucosyl transferase TCH4. *TCH* is related to plant perception of external stimuli and morphogenesis during adaptation. *TCH* is up-regulated when plants are stimulated by environmental stimuli [94]. However, the differential genes that correspond to *TCH* were down-regulated under salt stress in this experiment.

*CRE1* has been identified as a cytokinin receptor in *A. thaliana* [95]. Cytokinin is an important plant hormone regulating cell division and differentiation [96]. In response to abiotic stress, plants may repair tissue damage, which may require cytokinins. *SaAHK4* was found to be expressed both under alkali and drought stress, corresponding to the *CRE1* metabolic pathway and the expression level was similar between alkali and drought treatments. *CTR1* encodes a member of the protein kinase Raf family, and down-regulates the ethylene response pathway in *A. thaliana* [97]. There have been some reports about *CTR1* involvement in stress responses. For instance, the expression of *GmCTR1* in soybean infected with scab was up-regulated, peaked 48 h after infection, and subsequently decreased slightly; this indicates that *CTR1* might have a role in stress-responses [98]. *CTR1* overexpression in tobacco can make transgenic tobacco more sensitive to salt stress, suggesting that *CTR1* may down-regulate responses to salt stress [99]. The study of Arabidopsis *ctr1* mutants showed that Ctr1 may reduce the salt tolerance of Arabidopsis through negative regulation of ethylene signal [100–104]. Our results showed that nine genes corresponding to *CTR1* were down-regulated under drought stress, which is consistent with previous findings about responses to abiotic stress processes. However, under alkali stress, we found that 17 up-regulated DEGs corresponded to *CTR1*. This indicates that *S. alopecuroides* uses different processes to cope with alkali and drought stress. Further, this analysis provides a new approach for studying the role of the *CTR1* gene in alkali-stress responses in *S. alopecuroides*.

Under stress, plant tissues produce ROS, which can destroy membrane structure, damage biological macromolecules, cause metabolic disorders, and in severe cases cause plant death [105–110]. Superoxide dismutase, peroxidase, and catalase are protective enzymes in plants. Their main functions are to scavenge ROS free radicals, prevent their excessive accumulation, avoid or mitigate ROS attack on membranes, and prevent membrane damage [106, 111, 112]. In the KEGG enrichment pathway sequences, our salt, alkali, and drought treatments all reflected the presence of superoxide dismutase, peroxidase, and catalase. Through screening and comparison, we found some DEGs, including *SaPXR1, SaSOD1, SaSOD2–1, SaSOD2–2,* and *SaHACL1,* which code for superoxide dismutase, peroxidase, and catalase. This suggests that the physiological and biochemical processes and regulatory mechanisms differ depending on the type of abiotic stress.

## Conclusions

Our study is the first to analyze DEG expression using no-reference transcriptome sequencing under salt and alkali drought stress in *S. alopecuroides*. We have identified many candidate functional genes that are involved in multiple salt, alkali, and drought stress tolerance mechanisms, and these should be studied further. We found that key pathways, such as those response to plant hormone signal transduction, biosynthesis of secondary metabolites, metabolic processes, peroxisome production, and cellular morphology, were involved in abiotic-stress tolerance in this species*.* Many genes (DEGs) were expressed differentially between the treatments and the control. Our findings may assist in molecular design breeding about molecular adaptations to extreme environment and will provide resources for the study of plant stress resistance.

## Methods

### Plant materials and treatment

The 10 g seeds of *S. alopecuroides* were collected from Korla region, Xinjiang Autonomous Region, China (41° 45′ N, 86° 8′ E). After treatment using 98% H_2_SO_4_, the seeds were dipped in sterile water for 12 h and planted in pots filled with soil and vermiculite (1:1), and kept in day/night of 16 h/8 h, 25 °C /22 °C. Four-week-old seedlings were divided into 4 groups: control (CK), salt treatment (S_T), alkali treatment (AS_T) and drought treatment (DT), and each treatment was replicated three times. For the salt, alkali, and drought treatments, 1.2% NaCl, 1.2% NaHCO_3_ and 8% PEG6000, respectively, were added to 1/8th strength Hoagland’s nutrient solution. After 72 h of treatment, the root tissues of the control group and the treatment group were taken, and stored at − 80 °C.

### RNA extraction, quantification, and qualification

The extraction and isolation of RNA used the previous methods [57], with 3 replicates for each group. Then use the 2100 bioanalytical instrument to evaluate the quality of the obtained RNA.

### cDNA library preparation for transcription sequencing

Following the manufacturer’s recommendations of RNA Library Preparation Kit (New England Biolab, Massachusetts, USA), a sequencing library was constructed. Magnetic beads with poly-T oligonucleotides attached were used to enrich the mRNA. At a high temperature, divalent cations were used to carry out the cleavage. First strand of cDNA was synthesized using random hexamer primers. cDNA library was prepared followed the previous research [113]. The library was estimated and sequenced on the Illumina HiSeq platform (New England BioLabs, MA, USA) [113].

### Quality control, assembly, and annotation of unigenes

Adapter, ply-N and reads with low quality were removed to generated clean data, which was performed in downstream analysis. Transcriptome was assembed using Trinity by Beijing Novogene Company with reference to previous research methods [114]. Annotation of gene function using the BLASTX algorithm (E value < 1.0 E^− 5^) is based on the following databases: Nt, Nr, GO, Pfam, Swiss-Prot, KOG/COG, and KO. To obtain effective unigenes, all assembled unigenes were screened against multiple databases. Using Nr annotation, unigenes were assigned to BP, MF, and CC ontologies according to the GO framework using Blast2GO software [115]. Pathway distributions were perfomed on the basis of KEGG database using the BLASTX algorithm (E value < 1.0 E^− 5^).

### Analysis of differentially expressed genes (DEGs)

The transcriptome obtained using the Trinity platform provided a reference sequence (hereafter “ref”). RSEM software was used to map clean reads for every sample on ref. [116]. The results were calculated using RSEM, and the read count of each gene was procured. FPKM was used to determine the number and expression level for each unigene. The input data of DEG was the read count data obtained from the analysis of gene expression levels [117]. We used the DESeq method to analyze three biological replicate samples, and the screening threshold was padj < 0.05 [118]. Negative binomial models were used to calculate the p-value. GO and KEGG pathway enrichment analysis were used to analyze the DEGs. GOseq^5^ GO enrichment analysis method is based on Wallenius’ noncentral hypergeometric distribution [115]. KOBAS software was used to examin the effective enrichment of KEGG pathway [119].

### Verification of sequencing results by real-time quantitative RT-PCR

To verify the reliability of the sequencing results, 24 unigenes were randomly selected for expression analysis. Total RNA (5 μg) from each of three replicates was extracted and the primers were listed in Table S4. To perform qRT-PCR, 20 μL of reaction mixture was placed in a 96-well plate in the StepOnePlus™ Real-Time PCR System using SYBR *Premix Ex Taq*™ II kit. Each sample had three independent biological and technical replications. The expression level of the actin gene (J01298) was used to normalize target gene expression. Relative transcription levels were calculated using the double delta CT (2^−ΔΔCt^) method.

## Supplementary information

**Additional file 1: Figure S1.** Phenotypes of *Sophora alopecuroides* at different concentration in salt, alkali and PEG. This figure provides the phenotype under salt, alkali and drought treatment (The figures were taken by authors themselves).

**Additional file 2: Table S1.** Statistics of the number of DEGs corresponding to KEGG metabolic pathways. This table provides the number of DEGs corresponding to KEGG metabolic pathways.

**Additional file 3: Table S2.** Statistics of DEGs corresponding to KEGG metabolic pathways. This table provides the potential genes corresponding to KEGG metabolic pathways.

**Additional file 4: Table S3.** Statistics of the number of transcription factors corresponding to each transcription factor family. This table provides the number of transcription factors corresponding to each transcription factor family.

**Additional file 5: Table S4.** Primer sequences validated by qRT-PCR in this study. This table provides the primer sequences that were validated by qRT-PCR in this study.

## Data Availability

Raw data was deposited in NCBI database under SRA accession: PRJNA636118 (https://www.ncbi.nlm.nih.gov/sra/PRJNA636118).

## Abbreviations

ABA: Abscisic acid
DEG: Differentially expressed gene
COG: Clusters of Orthologous Groups of proteins
GO: Gene Ontology
KEGG: Kyoto Encyclopedia of Genes and Genomes
KOG: EuKaryotic Ortholog Groups
Nr: NCBI non-redundant protein Sequences
Nt: NCBI nucleotide Sequences
Pfam: Protein family
Swiss-Prot: Manually annotated and reviewed protein sequence database

## References

[1] Dagar JC, Sharma PC, Chaudhari SK, Jat HS, Sharif A (2016). Climate change Vis-a-Vis saline agriculture: impact and adaptation strategies.

[2] Kang JJ, Zhao WZ, Ming Z, Zheng Y, Yang F (2015). NaCl and Na_2_SiO_3_ coexistence strengthens growth of the succulent xerophyte Nitraria tangutorum under drought. Plant Growth Regul.

[3] FAO/AGL Extent and causes of salt-affected soils in participating countries. FAO/AGL- global network on integrated soil management for sustainable use of salt-affected lands. 2000. http://www.fao.org/ag/agl/agll/spush/topic2.htm.

[4] Dasgupta S, Hossain MM, Huq M, Wheeler D (2018). Climate change, salinization and high-yield rice production in coastal Bangladesh. Agric Resourc Econ Rev.

[5] Mandal AK, Sharma RC, Singh G, Dagar JC. Computerized database on salt affected soils in India. Technical bulletin No.2/2010.

[6] Zhao K, Song J, Feng G, Zhao M, Liu J (2011). Erratum to: species, types, distribution, and economic potential of halophytes in China. Plant Soil.

[7] Qadir M, Tubeileh A, Akhtar J, Larbi A, Minhas PS, Khan MA (2008). Productivity enhancement of salt-affected environments through crop diversification. Land Degrad Dev.

[8] Flowers TJ, Galal HK, Bromham L (2010). Evolution of halophytes: multiple origins of salt tolerance in land plants. Funct Plant Biol.

[9] Goñi O, Quille P, O’Connell S (2018). Ascophyllum nodosum extract biostimulants and their role in enhancing tolerance to drought stress in tomato plants. Plant Physiol Biochem.

[10] Harrison MT, Tardieu F, Dong Z, Messina CD, Hammer GL (2014). Characterizing drought stress and trait influence on maize yield under current and future conditions. Glob Chang Biol.

[11] Shanker AK, Maheswari M, Yadav SK, Desai S, Bhanu D, Attal NB, Venkateswarlu B (2014). Drought stress responses in crops. Funct Integr Genomics.

[12] Golldack D, Li C, Mohan H, Probst N (2014). Tolerance to drought and salt stress in plants: unraveling the signaling networks. Front Plant Sci.

[13] Hong Y, Zhao J, Guo L, Kim SC, Deng X, Wang G, Zhao G, Li M, Wang X (2016). Plant phospholipases D and C and their diverse functions in stress responses. Prog Lipid Res.

[14] Zhu J, Jianping NI (2016). Abiotic stress signaling and responses in plants. Cell..

[15] Hauser F, Li Z, Waadt R, Schroeder JI (2017). SnapShot: Abscisic acid signaling. Cell..

[16] Li L, Li M, Qi X, Tang X, Zhou Y (2018). De novo transcriptome sequencing and analysis of genes related to salt stress response in *Glehnia littoralis*. PeerJ.

[17] Kumar P, Pathania S, Katoch P (2006). Comparative EST profiles of leaf and root of Leymus chinensis, a xerophilous grass adapted to high pH sodic soil. Plant Sci.

[18] Wang W, Vinocur B, Altman A (2003). Plant responses to drought, salinity and extreme temperatures: towards genetic engineering for stress tolerance. Planta (Berlin).

[19] Tian DQ, Pan XY, Yu YM, Wang WY, Zhang F, Ge YY, Shen XL, Shen FQ, Liu XJ (2013). De novo characterization of the Anthurium transcriptome and analysis of its digital gene expression under cold stress. BMC Genomics.

[20] Long Y, Zhang J, Tian X, Wu S, Zhang Q, Zhang J, Dang Z, Pei XW (2014). De novo assembly of the desert tree Haloxylon ammodendron (C. A. Mey.) based on RNA-Seq data provides insight into drought response, gene discovery and marker identification. BMC Genomics.

[21] Yan J, Yu L, Xuan J, Lu Y, Lu S, Zhu W (2016). De novo transcriptome sequencing and gene expression profiling of spinach (*Spinacia oleracea* L.) leaves under heat stress. Sci Rep.

[22] Higashi Y, Okazaki Y, Myouga F, Shinozaki K, Saito K (2015). Landscape of the lipidome and transcriptome under heat stress in Arabidopsis thaliana. Sci Rep.

[23] Wu J, Zhang Y, Zhang H, Huang H, Folta Kevin M, Lu J (2010). Whole genome wide expression profiles ofVitis amurensisgrape responding to downy mildew by using Solexa sequencing technology. BMC Plant Biol.

[24] Zhou Y, Gao F, Liu R, Feng J, Li H (2012). De novo sequencing and analysis of root transcriptome using 454 pyrosequencing to discover putative genes associated with drought tolerance in Ammopiptanthus mongolicus. BMC Genomics.

[25] Qi XH, Xu XW, Lin XJ, Zhang WJ, Chen XH (2012). Identification of differentially expressed genes in cucumber (Cucumis sativus L.) root under waterlogging stress by digital gene expression profile. Genomics..

[26] Wang G, Zhu Q, Meng Q, Wu C (2012). Transcript profiling during salt stress of young cotton (Gossypium hirsutum) seedlings via Solexa sequencing. Acta Physiol Plant.

[27] Matsui A, Ishida J, Morosawa T, Mochizuki Y, Kaminuma E, Endo TA, Okamoto M, Nambara E, Nakajima M, Kawashima M, Satou M, Kim JM, Kobayashi N, Toyoda T, Shinozaki K, Seki M (2008). Arabidopsis transcriptome analysis under drought, cold, high-salinity and ABA treatment conditions using a tiling array. Plant Cell Physiol..

[28] Higashi Y, Okazaki Y, Myouga F, Shinozaki K, Saito K (2015). Landscape of the lipidome and transcriptome under heat stress inArabidopsis thaliana. Sci Rep.

[29] Aihua L, Zhaojun S (2000). The developmental situation and application potential of Sophara alopecuroides L. J Ningxia Univ.

[30] Zhao LF, Xu YJ, Ma ZQ, Deng ZS, Shan CJ, Wei GH (2013). Colonization and plant growth promoting characterization of endophytic Pseudomonas chlororaphis strain Zong1 isolated from Sophora alopecuroides root nodules. Braz J Microbiol.

[31] Begley T, Kinsland C, Strauss E (2001). The biosynthesis of coenzyme a in bacteria. Vitam Horm.

[32] Rubio S, Whitehead L, Larson T, Graham I, Rodriguez P (2008). The coenzyme a biosynthetic enzyme phosphopantetheine adenylyltransferase plays a crucial role in plant growth, salt/osmotic stress resistance, and seed lipid storage. Plant Physiol.

[33] Wang J, Jiang X, Zhao C, Fang Z, Jiao P (2020). Transcriptomic and metabolomic analysis reveals the role of CoA in the salt tolerance of Zygophyllum spp. BMC Plant Biol.

[34] Zhao Y, Chan Z, Gao J, Xing L, Cao M, Yu C, Hu Y, You J, Shi H, Zhu Y, Gong Y, Mu Z, Wang H, Deng X, Wang P, Bressan RA, Zhu JK (2016). ABA receptor PYL9 promotes drought resistance and leaf senescence. Proc Natl Acad Sci U S A.

[35] Craddock CP, Adams N, Kroon JT, Bryant FM, Hussey PJ, Kurup S, Eastmond PJ (2017). Cyclin-dependent kinase activity enhances phosphatidylcholine biosynthesis in Arabidopsis by repressing phosphatidic acid phosphohydrolase activity. Plant J.

[36] Ren R, Wei Y, Ahmad S, Jin J, Gao J, Lu C, Zhu G, Yang F. Identification and characterization of NPR1 and PR1 homologs in Cymbidium orchids in response to multiple hormones, salinity and viral stresses. Int J Mol Sci. 2020;21(6):1977. 10.3390/ijms21061977 PMC7139473 32183174

[37] Seo SY, Wi SJ, Park KY (2020). Functional switching of NPR1 between chloroplast and nucleus for adaptive response to salt stress. Sci Rep.

[38] Huang P, Dong Z, Guo P, Zhang X, Qiu Y, Li B, Wang Y, Guo H (2020). Salicylic acid suppresses apical hook formation via NPR1-mediated repression of EIN3 and EIL1 in Arabidopsis. Plant Cell.

[39] Wu Z, Han S, Zhou H, Tuang ZK, Wang Y, Jin Y, Shi H, Yang W (2019). Cold stress activates disease resistance in Arabidopsis thaliana through a salicylic acid dependent pathway. Plant Cell Environ.

[40] Li R, Liu C, Zhao R, Wang L, Chen L, Yu W, Zhang S, Sheng J, Shen L (2019). CRISPR/Cas9-mediated SlNPR1 mutagenesis reduces tomato plant drought tolerance. BMC Plant Biol.

[41] Khan A, Kamran M, Imran M, Al-Harrasi A, Al-Rawahi A, Al-Amri I, Lee IJ, Khan AL (2019). Silicon and salicylic acid confer high-pH stress tolerance in tomato seedlings. Sci Rep.

[42] Liu X, Zhao C, Gong Q, Wang Y, Cao J, Li X, Grierson D, Sun C. Characterization of a caffeoyl-CoA O-methyltransferase-like enzyme involved in biosynthesis of polymethoxylated flavones in Citrus reticulata. J Exp Bot. 2020;71(10):3066–79. 10.1093/jxb/eraa083 PMC7475179 32182355

[43] Robert HS, Grunewald W, Sauer M, Cannoot B, Soriano M, Swarup R, Weijers D, Bennett M, Boutilier K, Friml J (2015). Plant embryogenesis requires AUX/LAX-mediated auxin influx. Development..

[44] Swarup R, Bhosale R (2019). Developmental roles of AUX1/LAX Auxin influx carriers in plants. Front Plant Sci.

[45] Pulungan SI, Yano R, Okabe Y, Ichino T, Kojima M, Takebayashi Y, Sakakibara H, Ariizumi T, Ezura H (2018). SlLAX1 is required for Normal leaf development mediated by balanced Adaxial and Abaxial pavement cell growth in tomato. Plant Cell Physiol..

[46] Zhang C, Dong W, Huang ZA, Cho M, Yu Q, Wu C, Yu C (2018). Genome-wide identification and expression analysis of the CaLAX and CaPIN gene families in pepper (Capsicum annuum L.) under various abiotic stresses and hormone treatments. Genome..

[47] Kieber JJ, Schaller GE. Cytokinin signaling in plant development. Development. 2018;145(4):dev149344. 10.1242/dev.149344 29487105

[48] Arnaud D, Lee S, Takebayashi Y, Choi D, Choi J, Sakakibara H, Hwang I (2017). Cytokinin-mediated regulation of reactive oxygen species homeostasis modulates Stomatal immunity in Arabidopsis. Plant Cell.

[49] Wang B, Chen Y, Guo B, Kabir MR, Yao Y, Peng H, Xie C, Zhang Y, Sun Q, Ni Z (2014). Mol. Expression and functional analysis of genes encoding cytokinin receptor-like histidine kinase in maize (Zea mays L.). Genet Genomics.

[50] Verma V, Ravindran P, Kumar PP (2016). Plant hormone-mediated regulation of stress responses. BMC Plant Biol.

[51] Nejat N, Mantri N (2017). Plant immune system: crosstalk between responses to biotic and abiotic stresses the missing link in understanding plant Defence. Curr Issues Mol Biol.

[52] Bielach A, Hrtyan M, Tognetti VB. Plants under stress: involvement of Auxin and Cytokinin. Int J Mol Sci. 2017;18(7):1427. 10.3390/ijms18071427 PMC5535918 28677656

[53] Del Río LA, López-Huertas E (2016). ROS generation in peroxisomes and its role in cell signaling. Plant Cell Physiol.

[54] Liu L, Li J (2019). Communications between the endoplasmic reticulum and other organelles during abiotic stress response in plants. Front Plant Sci.

[55] Wang P, Zhao Y, Li Z, Hsu CC, Liu X, Fu L, Hou YJ, Du Y, Xie S, Zhang C, Gao J, Cao M, Huang X, Zhu Y, Tang K, Wang X, Tao WA, Xiong Y, Zhu JK (2018). Reciprocal Regulation of the TOR Kinase and ABA Receptor Balances Plant Growth and Stress Response. Mol Cell.

[56] Pan J, Li Z, Wang Q, Yang L, Yao F, Liu W (2020). An S-domain receptor-like kinase, OsESG1, regulates early crown root development and drought resistance in rice. Plant Sci.

[57] Su LT, Li JW, Liu DQ (2014). A novel MYB transcription factor, GmMYBJ1, from soybean confers drought and cold tolerance in Arabidopsis thaliana. Gene.

[58] Lian-Tai S, Wang Y, Liu D-Q, Li X-W, Zhai Y, Sun X, Li X-Y, Liu Y-J, Li J-W, Wang Q-Y (2015). The soybean gene, GmMYBJ2,encodes a R2R3-type transcription factor involved in drought stress tolerance in *Arabidopsis thaliana*. Acta Physiol Plant.

[59] Li XW, Wang Y, Yan F, et al. Overexpression of soybean R2R3-MYB transcription factor, GmMYB12B2, and tolerance to UV radiation and salt stress in transgenic Arabidopsis. Genet Mol Res Gmr. 2016;15(2):gmr.15026573. 10.4238/gmr.15026573 27323089

[60] He Y, Yang X, Xu C, Guo D, Niu L, Wang Y, Li J, Yan F, Wang Q (2018). Overexpression of a novel transcriptional repressor GmMYB3a negatively regulates salt-alkali tolerance and stress-related genes in soybean. Biochem Biophys Res Commun.

[61] He Y, Dong Y, Yang X (2020). Functional activation of a novel R2R3-MYB protein gene, GmMYB68, confers salt-alkali resistance in soybean ( Glycine max, L.). Genome..

[62] Misra P, Pandey A, Tiwari M, Chandrashekar K, Sidhu OP, Asif MH, Chakrabarty D, Singh PK, Trivedi PK, Nath P (2010). Modulation of Transcriptome and Metabolome of tobacco by Arabidopsis transcription factor, AtMYB12, Leads to Insect Resistance. Plant Physiol.

[63] Pandey A, Misra P, Khan MP, Swarnkar G, Tewari MC, Bhambhani S, Trivedi R, Chattopadhyay N, Trivedi PK (2014). Co-expression of Arabidopsis transcription factor, AtMYB12, and soybean isoflavone synthase, GmIFS1, genes in tobacco leads to enhanced biosynthesis of isoflavones and flavonols resulting in osteoprotective activity. Plant Biotechnol J.

[64] Wang F, Kong W, Wong G, Fu L, Peng R, Li Z, Yao Q (2016). AtMYB12 regulates flavonoids accumulation and abiotic stress tolerance in transgenic Arabidopsis thaliana. Mol Genet Genomics.

[65] Baloglu MC, Eldem V, Hajyzadeh M, Unver T (2014). Genome-wide analysis of the bZIP transcription factors in cucumber. PLoS One.

[66] Wei K, Chen J, Wang Y, Chen Y, Chen S, Lin Y, Pan S, Zhong X, Xie D (2012). Genome-wide analysis of bZIP-encoding genes in maize. DNA Res.

[67] Wang Z, Cheng K, Wan L, Yan L, Jiang H, Liu S, Lei Y, Liao B (2015). Genome-wide analysis of the basic leucine zipper (bZIP) transcription factor gene family in six legume genomes. BMC Genomics.

[68] Finkelstein RR, Gampala SS, Rock CD (2002). Abscisic acid signaling in seeds and seedlings. Plant Cell.

[69] Kim JS, Yamaguchi-Shinozaki K, Shinozaki K (2018). ER-anchored transcription factors bZIP17 and bZIP28 regulate root elongation. Plant Physiol.

[70] Satapathy L, Kumar D, Kumar M, Mukhopadhyay K (2018). Functional and DNA-protein binding studies of WRKY transcription factors and their expression analysis in response to biotic and abiotic stress in wheat (*Triticum aestivum* L.). 3 Biotech.

[71] Wu J, Chen J, Wang L, Wang S (2017). Genome-wide investigation of WRKY transcription factors involved in terminal drought stress response in common bean. Front Plant Sci.

[72] Liang MH, Jiang JG (2017). Analysis of carotenogenic genes promoters and WRKY transcription factors in response to salt stress in Dunaliella bardawil. Sci Rep.

[73] Zhao N, He M, Li L, Cui S, Hou M, Wang L, Mu G, Liu L, Yang X (2020). Identification and expression analysis of WRKY gene family under drought stress in peanut (*Arachis hypogaea* L.). PLoS One.

[74] Yang Z, Chi X, Guo F, Jin X, Luo H, Hawar A, Chen Y, Feng K, Wang B, Qi J, Yang Y, Sun B (2020). SbWRKY30 enhances the drought tolerance of plants and regulates a drought stress-responsive gene, SbRD19, in sorghum. J Plant Physiol.

[75] Zhou S, Zheng WJ, Liu BH, Zheng JC, Dong FS, Liu ZF, Wen ZY, Yang F, Wang HB, Xu ZS, Zhao H, Liu YW. Characterizing the role of TaWRKY13 in salt tolerance. Int J Mol Sci. 2019;20(22):5712. 10.3390/ijms20225712 PMC6888956 31739570

[76] Duan AQ, Yang XL, Feng K, Liu JX, Xu ZS, Xiong AS (2020). Genome-wide analysis of NAC transcription factors and their response to abiotic stress in celery (Apium graveolens L.). Comput Biol Chem.

[77] Sun H, Hu M, Li J, Chen L, Li M, Zhang S, Zhang X, Yang X (2018). Comprehensive analysis of NAC transcription factors uncovers their roles during fiber development and stress response in cotton. BMC Plant Biol.

[78] Duan M, Zhang R, Zhu F, Zhang Z, Gou L, Wen J, Dong J, Wang T (2017). A lipid-anchored NAC transcription factor is translocated into the nucleus and activates glyoxalase I expression during drought stress. Plant Cell.

[79] Hu H, Dai M, Yao J, Xiao B, Li X, Zhang Q, Xiong L (2006). Overexpressing a NAM, ATAF, and CUC (NAC) transcription factor enhances drought resistance and salt tolerance in rice. Proc Natl Acad Sci U S A.

[80] Jeong JS, Kim YS, Baek KH, Jung H, Ha SH, Do Choi Y, Kim M, Reuzeau C, Kim JK (2010). Root-specific expression of OsNAC10 improves drought tolerance and grain yield in Rice under field drought conditions. Plant Physiol.

[81] Shen H, Liu C, Zhang Y, Meng X, Zhou X, Chu C, Wang X (2012). OsWRKY30 is activated by MAP kinases to confer drought tolerance in rice. Plant Mol Biol.

[82] Wang CT, Ru JN, Liu YW, Li M, Zhao D, Yang JF, Fu JD, Xu ZS. Maize WRKY Transcription Factor ZmWRKY106 confers drought and heat tolerance in transgenic plants. Int J Mol Sci. 2018;19(10). 10.3390/ijms19103046. 10.3390/ijms19103046 PMC6213049 30301220

[83] Sakuraba Y, Kim YS, Han SH, Lee BD, Paek NC (2015). The Arabidopsis transcription factor NAC016 promotes drought stress responses by repressing AREB1 transcription through a trifurcate feedforward regulatory loop involving NAP. Plant Cell.

[84] Hao YJ, Wei W, Song QX, Chen HW, Zhang YQ, Wang F, Zou HF, Lei G, Tian AG, Zhang WK, Ma B, Zhang JS, Chen SY (2011). Soybean NAC transcription factors promote abiotic stress tolerance and lateral root formation in transgenic plants. Plant J.

[85] Chen Y, Liu ZH, Feng L, Zheng Y, Li DD, Li XB (2013). Genome-wide functional analysis of cotton (Gossypium hirsutum) in response to drought. PLoS One.

[86] Zhang F, Zhu G, Du L, Shang X, Cheng C, Yang B, Hu Y, Cai C, Guo W (2016). Genetic regulation of salt stress tolerance revealed by RNA-Seq in cotton diploid wild species, Gossypium davidsonii. Sci Rep.

[87] Li S, Fan C, Li Y, Zhang J, Sun J, Chen Y, Tian C, Su X, Lu M, Liang C, Hu Z (2016). Effects of drought and salt-stresses on gene expression in Caragana korshinskii seedlings revealed by RNA-seq. BMC Genomics.

[88] Muthusamy M, Uma S, Backiyarani S, Saraswathi MS, Chandrasekar A (2016). Transcriptomic changes of drought-tolerant and sensitive Banana cultivars exposed to drought stress. Front Plant Sci.

[89] Li CY, Deng GM, Yang J, Viljoen A, Jin Y, Kuang RB, Zuo CW, Lv ZC, Yang QS, Sheng O, Wei YR, Hu CH, Dong T, Yi GJ (2012). Transcriptome profiling of resistant and susceptible Cavendish banana roots following inoculation with Fusarium oxysporum f. sp. cubense tropical race 4. BMC Genomics.

[90] Raghavendra AS, Gonugunta VK, Christmann A, Grill E (2010). ABA perception and signalling. Trends Plant Sci.

[91] Ton J, Flors V, Mauch-Mani B (2009). The multifaceted role of ABA in disease resistance. Trends Plant Sci.

[92] Lechner E, Leonhardt N, Eisler H, Parmentier Y, Alioua M, Jacquet H, Leung J, Genschik P (2011). MATH/BTB CRL3 receptors target the Homeodomain-Leucine zipper ATHB6 to modulate Abscisic acid signaling. Dev Cell.

[93] Nishimura N, Hitomi K, Arvai AS, Rambo RP, Hitomi C, Cutler SR, Schroeder JI, Getzoff ED (2009). Structural mechanism of abscisic acid binding and signaling by dimeric PYR1. Science..

[94] Xu W, Purugganan MM, Polisensky DH, Antosiewicz DM, Fry SC, Braam J (1995). Arabidopsis TCH4, regulated by hormones and the environment, encodes a xyloglucan endotransglycosylase. Plant Cell.

[95] Inoue T, Higuchi M, Hashimoto Y, Seki M, Kobayashi M, Kato T, Tabata S, Shinozaki K, Kakimoto T (2001). Identification of CRE1 as a cytokinin receptor from Arabidopsis. Nature..

[96] Holst K, Schmülling T, Werner T (2011). Enhanced cytokinin degradation in leaf primordia of transgenic Arabidopsis plants reduces leaf size and shoot organ primordia formation. J Plant Physiol.

[97] Kieber JJ, Rothenberg M, Roman G, Feldmann KA, Ecker JR (1993). CTR1, a negative regulator of the ethylene response pathway in Arabidopsis, encodes a member of the Raf family of protein kinases. Cell..

[98] Zhang NN, Xue D, Cui XX, Zhao JM, Guo N, Wang HT, Xing H. Cloning and functional analysis of the CTR1 in soybean. Sci Agric Sin. 2017. 10.3864/j.issn.0578-1752.2017.16.003.

[99] Cai-Li BI, Wen XJ, Zhang XY, Xu L (2010). Cloning and characterization of a putative CTR1 gene from wheat. AGR Sci China.

[100] Kazan K (2015). Diverse roles of jasmonates and ethylene in abiotic stress tolerance. Trends Plant Sci.

[101] Jiang C, Belfield EJ, Cao Y, Smith JA, Harberd NP (2013). An Arabidopsis soil-salinity-tolerance mutation confers ethylene-mediated enhancement of sodium/potassium homeostasis. Plant Cell.

[102] Wang Y, Liu C, Li K, Sun F, Hu H, Li X, Zhao Y, Han C, Zhang W, Duan Y, Liu M, Li X (2007). Arabidopsis EIN2 modulates stress response through abscisic acid response pathway. Plant Mol Biol.

[103] Cao WH, Liu J, Zhou QY, Cao YR, Zheng SF, Du BX, Zhang JS, Chen SY (2010). Expression of tobacco ethylene receptor NTHK1 alters plant responses to salt stress. Plant Cell Environ.

[104] Achard P, Cheng H, De Grauwe L, Decat J, Schoutteten H, Moritz T, Van Der Straeten D, Peng J, Harberd NP (2006). Integration of plant responses to environmentally activated phytohormonal signals. Science..

[105] Anjum NA, Sofo A, Scopa A, Roychoudhury A, Gill SS, Iqbal M, Lukatkin AS, Pereira E, Duarte AC, Ahmad I (2015). Lipids and proteins—major targets of oxidative modifications in abiotic stressed plants. Environ Sci Pollut Res Int.

[106] Gill SS, Tuteja N (2010). Reactive oxygen species and antioxidant machinery in abiotic stress tolerance in crop plants. Plant Physiol Biochem.

[107] Hasanuzzaman M, Hossain MA, Silva JATD, Hasanuzzaman M, Hossain MA, Jaime A, da Silva Masayuki Fujita T (2012). Plant response and tolerance to abiotic oxidative stress: Antioxidant defense is a key factor.

[108] Noctor G, Gomez L, Vanacker H, Foyer CH (2002). Interactions between biosynthesis, compartmentation and transport in the control of glutathione homeostasis and signaling. J Exp Bot.

[109] Noctor G, Veljovic-Jovanovic S, Driscoll S, Novitskaya L, Foyer CH (2002). Drought and oxidative load in the leaves of C3 plants: a predominant role for photorespiration?. Ann Bot.

[110] Khan MN, Mobin M, Abbas ZK, AlMutairi KA, Siddiqui ZH (2017). Role of nanomaterials in plants under challenging environments. Plant Physiol Biochem.

[111] Alscher RG, Erturk N, Heath LS (2002). Role of superoxide dismutases (SODs) in controlling oxidative stress in plants. J Exp Bot.

[112] Garg N, Manchanda G (2009). ROS generation in plants: boon or bane?. G Bot Ital.

[113] Yuan W, Liu Z, Lei W, Sun L, Yang H, Wang Y, Ramdas S, Dong X, Xu R, Cai H, Li JZ, Ke Y (2017). Mutation landscape and intra-tumor heterogeneity of two MANECs of the esophagus revealed by multi-region sequencing. Oncotarget..

[114] Grabherr MG, Haas BJ, Yassour M, Levin JZ, Thompson DA, Amit I, Adiconis X, Fan L, Raychowdhury R, Zeng Q, Chen Z, Mauceli E, Hacohen N, Gnirke A, Rhind N, di Palma F, Birren BW, Nusbaum C, Lindblad-Toh K, Friedman N, Regev A (2011). Full-length transcriptome assembly from RNA-Seq data without a reference genome. Nat Biotechnol.

[115] Young MD, Wakefield MJ, Smyth GK, Oshlack A (2010). Gene ontology analysis for RNA-SEQ: accounting for selection bias. Genome Biol.

[116] Bertioli DJ, Cannon SB, Froenicke L, Huang G, Farmer AD, Cannon EK, Liu X, Gao D, Clevenger J, Dash S, Ren L, Moretzsohn MC, Shirasawa K, Huang W, Vidigal B, Abernathy B, Chu Y, Niederhuth CE, Umale P, Araújo AC, Kozik A, Kim KD, Burow MD, Varshney RK, Wang X, Zhang X, Barkley N, Guimarães PM, Isobe S, Guo B, Liao B, Stalker HT, Schmitz RJ, Scheffler BE, Leal-Bertioli SC, Xun X, Jackson SA, Michelmore R, Ozias-Akins P (2016). The genome sequences of Arachis duranensis and Arachis ipaensis, the diploid ancestors of cultivated peanut. Nat Genet.

[117] Maza E, Frasse P, Senin P, Bouzayen M, Zouine M (2013). Comparison of normalization methods for differential gene expression analysis in RNA-Seq experiments: a matter of relative size of studied transcriptomes. Commun Integr Biol.

[118] Anders S, Huber W (2010). Differential expression analysis for sequence count data. Genome Biol.

[119] Mao X, Cai T, Olyarchuk JG, Wei L (2005). Automated genome annotation and pathway identification using the KEGG Orthology (KO) as a controlled vocabulary. Bioinformatics..

