# Supplementary material for: De novo transcriptome sequencing and analysis of salt-, alkali-, and drought-responsive genes in Sophora alopecuroides
Source: BMC Genomics. 2020 Jun 23;21:423. doi: 10.1186/s12864-020-06823-4 (PMC7310485; doi:10.1186/s12864-020-06823-4)
Supplement: Supplementary file 1 — Additional file 1: Figure S1. Phenotypes of Sophora alopecuroides at different concentration in salt, alkali and PEG. This figure provides the phenotype under salt, alkali and drought treatment (The figures were taken by authors themselves). [file 12864_2020_6823_MOESM1_ESM.pdf]

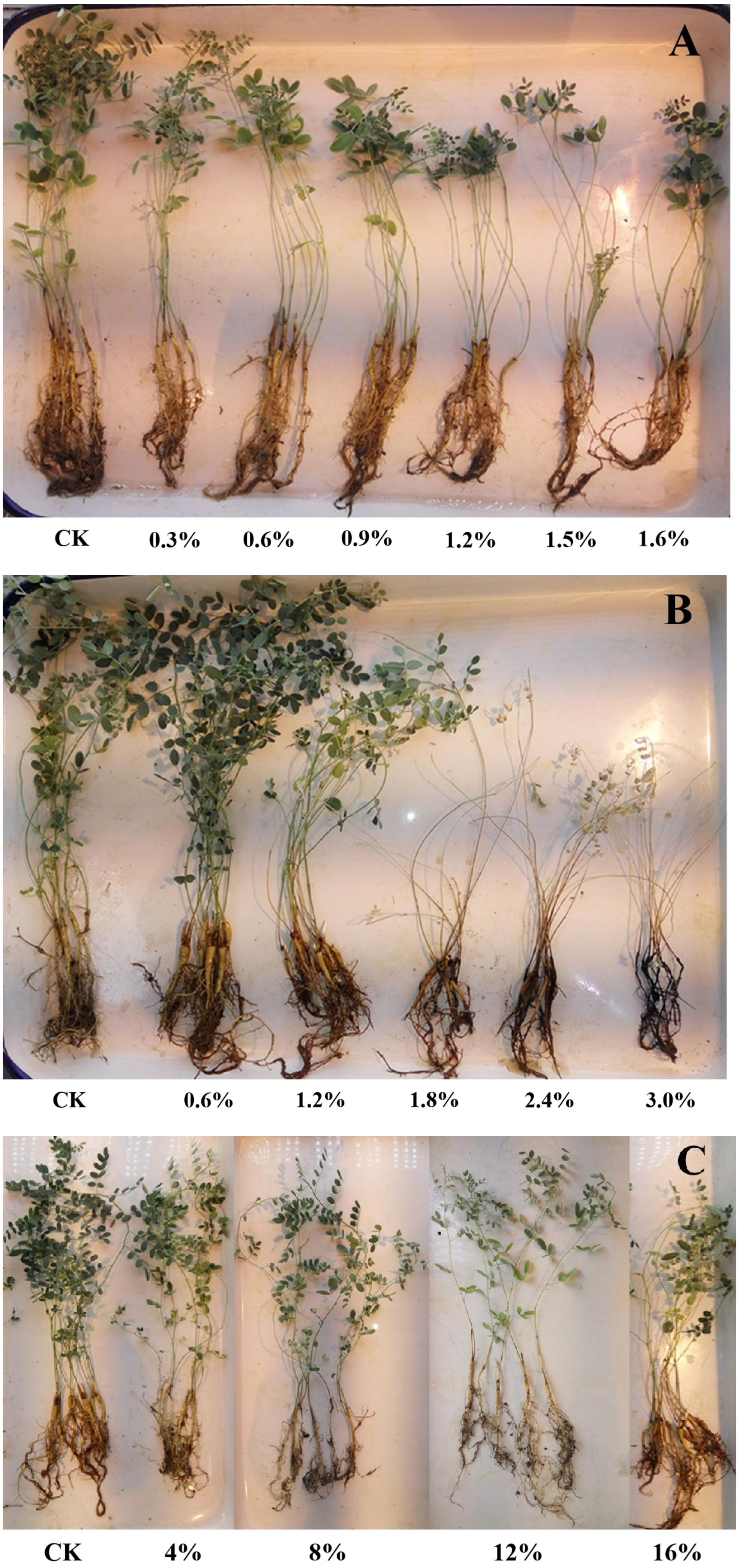

Figure S1. Phenotypes of *Sophora alopecuroides* at different concentration in salt, alkali and PEG

A：NaCl; B: $NaHCO_3$; C: PEG
